# The Potential Role of Liquid Biopsies in Advancing the Understanding of Neuroendocrine Neoplasms

**DOI:** 10.3390/jcm10030403

**Published:** 2021-01-21

**Authors:** Dinakshi Shah, Angela Lamarca, Juan W Valle, Mairéad G McNamara

**Affiliations:** 1 Department of Medical Oncology, The Christie NHS Foundation Trust, Manchester M20 4BX, UK; dinakshi.shah@nhs.net (D.S.); angela.lamarca@nhs.net (A.L.); 2 Division of Cancer Sciences, University of Manchester, Manchester M13 9PL, UK; juan.valle@nhs.net

**Keywords:** neuroendocrine tumours, neuroendocrine neoplasms, biomarkers, circulating tumour cells, cell free DNA, circulating tumour DNA, tumour nucleic acid, mRNA

## Abstract

Tumour tissue as a source for molecular profiling and for in vivo models has limitations (e.g., difficult access, limited availability, single time point, potential heterogeneity between primary and metastatic sites). Conversely, liquid biopsies provide an easily accessible approach, enabling timely and longitudinal interrogation of the tumour molecular makeup, with increased ability to capture spatial and temporal intra-tumour heterogeneity compared to tumour tissue. Blood-borne biomarker assays (e.g., circulating tumour cells (CTCs), circulating free/tumour DNA (cf/ctDNA)) pose unique opportunities for aiding in the molecular characterisation and phenotypic subtyping of neuroendocrine neoplasms and will be discussed in this article.

## 1. Introduction

Neuroendocrine neoplasms (NENs) are a rare and heterogeneous group of neoplasms with significant variation in presentation, prognosis and clinical course [1]. These malignancies can arise from almost any endocrine cell in the body but predominantly originate from the gastro-entero-pancreatic (GEP-NENs) or bronchopulmonary tract, making up 95% of diagnoses [2,3].

Hypersecretion of hormones and monoamines are a hallmark of NENs, and patients can present with symptoms of hormone overproduction, classified as functional [4]. Others can remain asymptomatic or have non-specific symptoms (non-functional), and due to this variation and the multisystem presentation, diagnosis can be delayed by up to four years [5]. NENs are classified by the World Health Organisation (WHO) 2017 grading system according to Ki-67 or mitotic index and cellular differentiation; Grade 1 tumours are well-differentiated with a Ki-67 < 3%, Grade 2 tumours are well-differentiated with a Ki-67 of 3–20% and Grade 3 neoplasms are further divided into well-differentiated tumours with a Ki-67 > 20% or poorly differentiated neuroendocrine carcinomas (NECs) [6]. Well-differentiated, low-proliferating tumours are termed neuroendocrine tumours (NETs).

The management of NENs is complex and variable depending on primary site, presence of metastatic disease and grade. In early-stage disease, patients may be eligible for surgery, however, for patients that present with disseminated disease, cure is not feasible [7]. For these patients, treatment options include somatostatin analogues (SSAs), chemotherapy, targeted therapy and peptide receptor radionuclide therapy (PRRT) [8]. Current management in Europe is based on guidelines produced by the European Neuroendocrine Tumour Society (ENETS), the European Society for Medical Oncology (ESMO) and multidisciplinary team (MDT) discussion [9].

The median survival for all patients with NENs is 42 months, although this can vary significantly amongst primary tumour sites and grade, with better prognosis in rectal and small bowel NETs and the highest mortality in patients with advanced poorly differentiated NECs [8]. Biological behaviour in NENs can also vary significantly, from indolent disease to highly aggressive cancers [10]. Given the distinct phenotypic variants and disease heterogeneity, there is a need for robust biomarkers to help determine prognosis, treatment selection and response.

The vast majority, 70–90%, of well-differentiated NETs tend to overexpress somatostatin receptors (SSTRs), which have been used as a specific marker in nuclear medicine imaging [11] ^68^Gallium DOTA SSTR peptide positron emission tracer computed tomography (^68^Ga PET-CT) has emerged as superior to other imaging modalities in identifying SSTRs and is useful in monitoring disease status and identifying new sites of disease. However, this is limited in tumours without SSTR2 expression and by lack of widespread availability of ^68^Ga PET-CT.

Chromogranin A (CgA) and urinary/serum 5-hydroxy-indoleacetic acid (5-HIAA) are commonly used as biomarkers in patients with NENs to monitor disease and treatment, in conjunction with imaging. Chromogranin A is secreted by most NENs and has been shown to be present in up to 90% of patients with GEP-NENs [12]. However, CgA has a specificity of less than 50%, and can be elevated in other conditions such as renal failure and cardiac disease, atrophic gastritis and proton pump inhibitor use [2]. 5-hydroxy-indoleacetic acid, a metabolite of serotonin, is measured as a marker of carcinoid syndrome and has a sensitivity of between 35–73%, and specificity between 89–100%, with limited prognostic value [13].

Biomarkers have several uses in clinical practice; Type 0 biomarkers are used to determine the natural history of the disease, Type I biomarkers can capture treatment efficacy and Type II biomarkers are considered surrogate endpoints for disease that predict clinical benefit [14,15]. A National Cancer Institute Neuroendocrine Tumour summit conference held in 2007 noted biomarker limitations to be a crucial unmet need to aid diagnosis, molecular characterisation and management in these cancers [16].

## 2. Liquid Biopsies

Liquid biopsies have gained increasing interest in the growing era of precision medicine. Liquid biopsies are a minimally invasive tool used to detect circulating biomarkers from blood or other bodily fluids to provide information on tumour biology or therapeutic response that is traditionally obtained through tissue biopsy or imaging [17].

Current medical practice for tissue diagnosis involves direct sampling, which may be invasive, can fail to capture heterogeneity of tumours and may require the need for repeat sampling if an inadequate specimen is obtained. Surveillance of patients with imaging such as computed tomography (CT) and magnetic resonance imaging (MRI) is expensive, time consuming and may not detect micro-metastases [18]. Blood-based assays for tumour diagnosis, screening and monitoring are therefore highly attractive, as they are faster to obtain, less invasive and can aid molecular profiling for therapeutic targets, monitor disease status and determine response to treatment in ‘real time.’ In addition, they can be cost effective by reducing the use of ineffective treatments.

Due to advances in technology for gene amplification and sequencing, liquid biopsies can access an increasing number of circulating biomarkers in cancer [17]. The study of liquid biopsies in other cancers have focused on the use of circulating tumour cells (CTCs) as a biomarker to determine cancer burden, and circulating free/tumour DNA (cf/ctDNA) and circulating transcript profiling for genomics analysis of tumours [19]. The integration of liquid biopsy platforms in NENs has been increasingly studied in recent years. This review will focus on the role of CTCs and circulating tumour nucleotides as tumour biomarkers in patients with NENs.

## 3. Circulating Tumour Cells

Circulating tumour cells are released into the circulatory system from primary tumours or their metastatic sites. Circulating tumour cells were first described in 1860, when they were isolated in the peripheral blood of a patient with metastatic cancer [20]. Their evaluation in studies has since revolved around the mechanisms of cancer metastasis. Evidence has shown an association between the presence of CTCs and cancer metastases. New technologies have been devised in recent years to enrich CTCs and subsequently isolate them from other cells [21,22].

The potential use of CTCs has been demonstrated in breast cancer, where studies have observed CTCs as an independent predictor of progression free survival (PFS) and overall survival (OS) in metastatic disease [23]. Data from a recent, large, pooled analysis of 2761 patients with metastatic breast cancer has shown that OS for patients who remained CTC negative following treatment initiation was 45.6 months (hazard ratio (HR), 1.38; 95% CI 1.16–1.64) compared to 17.87 months in patients who were persistently positive for CTCs (HR 3.06, 95% CI 2.63–3.56). Furthermore, patients who were CTC positive at baseline and subsequently negative following treatment initiation had improved OS, highlighting a potential role for CTCs in monitoring early response [24]. Similar results have been observed in the enumeration of CTCs in advanced metastatic colorectal cancer [25,26].

The CellSearch platform is the only method approved by the Food and Drug Administration (FDA) for detection of CTCs due to high sensitivity and specificity, and therefore is the most widely studied; however, some have used negative enrichment techniques, independent of epithelial markers [26]. This has resulted in limitations in direct comparisons of results between studies.

### 3.1. Potential Prognostic Ability of CTCs

One of the first published studies investigating the presence of CTCs in patients with a NEN diagnosis was by Khan et al., who looked at epithelial cell adhesion molecule (EpCAM) expression and CTC detection in patients with metastatic NENs [27]. CTCs were isolated using the CellSearch platform, an automated system that uses EpCAM to detect and enrich these cells. The epithelial cell adhesion molecule was previously thought not to be expressed in NENs, as their origin is widely accepted to be derived from neural crest cells rather than epithelial cells [27]. Khan et al. reported that EpCAM expression, however, was demonstrated in all ileal and pancreatic NENs and CTCs were detected in 43% of midgut NENs and 21% of pancreatic NENs [27]. Ninety five percent of patients who had progressive disease according to the Response Evaluation Criteria in Solid Tumors (RECIST) had detectable CTCs across all grades, compared to only 20% of patients with stable disease (*p* < 0.001) Their presence was also correlated with burden of liver metastases (*p* < 0.001). This study was important in highlighting the feasibility of CTC detection in patients with a NEN diagnosis through EpCAM expression and the association found between CTCs and progressive disease.

Khan et al. have further studied the prognostic relevance of CTCs in a prospective cohort of patients with histologically proven metastatic NENs. The majority of patients in the sample had grade 1 or 2 tumours, with 17% having grade 3 tumours. They found an optimal prognostic threshold of ≥1 CTC per 7.5 mL of blood, demonstrating a worse PFS and OS in this group (HRs, 6.6 and 8.0, respectively, *p* < 0.001) [28]. Other studies have supported an association between CTCs and poor outcomes in patients with NENs (summarised in Table 1). A study assessing the prognostic value of CTCs, before and during treatment, in a small sample of patients from Asia with NENs, found a CTC baseline detection rate of 94.3%, and baseline counts were independent prognostic factors for PFS (*p* = 0.015) and OS (*p* = 0.023) [29]. All patients in the group had a histological diagnosis of NET, with the majority (42.9%) of the sample having grade 3 disease. Circulating tumour cells <20 cells/mL at baseline, prior to starting treatment, were found to be associated with a longer PFS (*p* = 0.003) and OS (*p* = 0.008). Further studies have supported these findings, with a CTC detection rate of at least one CTC in 36–68% patients with NENs and association with changes in CTCs and OS with HRs between 4.13 to 6.6 [27,28,30,31].

Small studies evaluating CTC enumeration in patients with Merkel cell carcinoma (MCC) have demonstrated that CTCs are also detectable in this tumour type (40–97%) and their presence is associated with increased burden of disease [32,33]. A study by Blom et al. assessing CTCs in 32 patients with localised, nodal and distant MCC found that the median survival time for patients positive for CTCs was 10.5 months and had not yet been reached at 25.6 months for patients negative for CTCs [33]. Similar studies conducted in patients with limited and extensive stage small cell lung cancer (SCLC) have also highlighted the prognostic value of CTCs, demonstrating that baseline CTC number and changes in CTC numbers are independent prognostic factors for OS and PFS [34,35,36,37].

### 3.2. Potential Predictive Ability of CTCs during Treatment

In 2016, Khan et al. investigated the relevance of CTCs in assessing treatment response in patients with histologically-proven metastatic NENs. The majority of patients (81%) in the sample had grade 1 or 2 tumours and 60% of patients had at least one CTC detected. Sixty five percent of patients with a CTC count > 8 had radiological disease progression compared to 4% of patients with no detectable CTCs. Post treatment counts were compared to baseline in patients with radiological progression and it was reported that 60% of patients with progression had a dynamic change (reduction of less than 50% or any increase in CTCs) compared to 8% of patients without (0 or >50% reduction in CTCs) (*p* < 0.001). This suggests that CTC changes may be a surrogate marker for radiological response to treatment in NENs [30].

Similar results have been observed in patients with SCLC. A small multi-centre pilot study assessing changes in CTC count in patients with extensive stage SCLC treated with platinum and etoposide reported a substantial reduction in CTCs post-treatment in 15 patients with follow up CTC measurements (median reduction 97.4%) [40]. Another study found that copy number alterations from CTCs in patients with SCLC could correctly identify 83.3% of patients as either chemo-sensitive or chemo-refractory, demonstrating a significant difference in PFS between the two groups (*p* = 0.0166). This suggests that copy number aberrations may be of value in predicting response to therapy in this patient group [41].

Circulating tumour cells have also been investigated for their expression of targetable markers. Childs et al. assessed SSTR expression in CTCs in metastatic midgut, pancreatic or unknown primary NENs, as these are commonly overexpressed in this disease group [39]. The majority (74%) of patients had grade 1 or 2 tumours and 33% of patients had evidence of expression of either SSTR2 or SSTR5 in CTCs. Eighty seven percent of the overall population had SSTR positive disease on scans. This suggests that CTCs may be a useful biomarker for evaluating SSTR status, in conjunction with imaging.

Although CTC enumeration as a biomarker is attractive, there have been several limitations identified. Cut-off vales for CTCs vary in studies; most have assessed for ≥1 CTC in 7.5 mL in blood; however, other studies have assessed for >5 CTCs and >20 in blood, and there is no consensus on the optimal cut-off value [29]. Furthermore, detection rate in NENs is low and evidence, thus far, is limited in assessing the implications of clinical utility of CTCs as a biomarker in everyday practice.

### 3.3. CTC Derived Models

Patient-derived xenografts (PDX) are used in translational research to aid further understanding of tumour biology and potential drug development [42]. This involves extracting tumour tissue from either primary or metastatic sites of cancer and implanting these into immunodeficient mice [43]. However, tumour tissue as a source for in vivo models has limitations due to finite availability and potentially difficult access. CTC-derived eXplant (CDX) models can offer solutions to this by utilising isolated CTCs from easily accessible plasma samples of patients with malignancies.

Breast cancer CDX models have been established when metastasis-initiating cells (MICs) amongst primary luminal breast cancer CTCs resulted in the development of bone, lung and liver metastases in six mice, 6–12 months after implantation [44].

Circulating tumour cell-derived eXplant models are therefore of great interest and have been attempted in NENs. Faugeroux et al. generated the first prostate NEC CDX model by using EpCAM CTCs isolated from blood samples from 15 patients with advanced castrate-resistant prostate cancer (CRPC) [45]. The blood samples were processed via CellSearch and CTCs were implanted in mice (median 230 CTCs). The CTCs from one patient with a very high CTC count (19,988 CTCs) resulted in the development of a palpable tumour in the model within 165 days of implantation. Whole exome sequencing (WES) was performed on the diagnostic biopsy, CTCs and CDX model, demonstrating 5–30% overlap in mutations between the primary tumour biopsy and CDX. Furthermore, genetic drivers were identified (tumour protein 53 *(TP53)*, phosphatase and tensin homolog *(PTEN)*, and RB transcriptional corepressor 1 *(RB1)*), indicating transition to neuroendocrine (CRPC-NE) malignancy.

Circulating tumour cells used to devise CDX models can provide further understanding of tumour characterisation and offer the potential for drug development. There are very few CDX models reported to date in NENs and studies should further assess the use of CTCs to develop clinically relevant in vivo models.

## 4. Cell Free DNA (cfDNA) and Circulating Tumour DNA (ctDNA)

Circulating free DNA is genetic material, released by apoptotic and necrotic cells. Circulating tumour DNA is a fragment of cfDNA that is released from cancer cells into the circulation or other bodily fluids [17]. These ctDNA cells can be isolated to extract genetic information related to the primary tumour. This has been well highlighted in non-small cell lung cancer, where ctDNA can be used to observe epidermal growth factor receptor (EGFR) targetable mutations in the primary tumour, reducing the need for tissue sampling [46]. In patients with localised colon cancer, detection of ctDNA following surgery has been shown to correlate with cancer relapse (HR 17.56 (*p* = 0.0014), highlighting a potential role for disease monitoring [47].

A potential challenge in the use of ctDNA for molecular profiling in patients with a NEN diagnosis is the relative lack of driver mutations in comparison with other tumour types [48]. Alterations in tumour suppressor multiple endocrine neoplasia-1 (*MEN1*) have been widely studied in patients with pancreatic NETs; however, limited data is available in other tumour types. Alterations in ATM serine/threonine kinase *(ATM)*, death-domain-associated protein (*DAXX*) and *TP53* have also been reported [1,49]. Table 2 summarises the relevant available evidence on potential utility of ctDNA in patients with a NEN diagnosis.

Zakka et al. were among the first to perform a population-based study to characterise genetic alterations in patients with NENs using ctDNA [50]. Circulating tumour DNA next generation sequencing (NGS) was performed on 320 patients, with 52% pancreatic NET, 16% gastrointestinal NEC, 7% large cell lung NEC, 5% nasopharyngeal NEC and NEN not specified. Genomic alterations were identified in 87.5% of samples and the most common mutations were found in *TP53*, *KRAS*, *EGFR*, *PIK3CA*, *BRAF*, *MYC* and *CCNE1*, demonstrating that ctDNA analysis is feasible in this patient group. The study was performed as a retrospective analysis and clinical information from a de-identified database had some limitations as no information was available on whether samples were obtained before or after surgery/systemic therapy, and tissue samples were not available for comparison, as acknowledged by the authors.

Several case studies have demonstrated the use of ctDNA to aid detection of disease, treatment selection and monitoring response. Klempner et al. reported the changes in urinary ctDNA in response to treatment in two patients with advanced high grade colorectal NEN [55]. One patient was treated with neoadjuvant chemoradiotherapy with carboplatin and etoposide, followed by resection, and had residual tumour and positive margins. The patient developed rapid radiological progression on temozolomide and therefore tissue from the liver biopsy was evaluated for any alterations for targeted treatment. Another patient had a large locally advanced rectal NEN, initially treated with cisplatin and etoposide, then carboplatin/docetaxel, and subsequently developed progressive disease. The rectal tissue underwent genomic profiling. In both cases, BRAF^V600E^ mutations were reported and an alteration frequency of 9% was reported in the patient group. Following initiation of therapy for cases with BRAF- and MEK- targeted combinations, urinary BRAF^V600E^ ctDNA rapidly decreased and mirrored resolution of symptoms. In another case, ctDNA demonstrated an ALK translocation in a patient with atypical carcinoid tumour of the lung. They were subsequently treated with an ALK inhibitor, alectinib, and had a good response to treatment, with a 60% partial response in brain metastases [51]. Another patient with large-cell neuroendocrine carcinoma of the cervix was successfully treated with nivolumab and stereotactic body radiotherapy following genomic mutation analysis from ctDNA, suggesting high tumour mutation burden [54].

The CIRCAN-NEC pilot study investigated ctDNA mutations in the blood samples of 24 patients with a diagnosis of GEP NECs or NEC from an unknown primary, to assess the sensitivity of ctDNA in characterising genetic alterations, and their value in predicting response to chemotherapy. Preliminary results from the published abstract have demonstrated mutations in *TP53*, *RB1*, and *KRAS,* and some also had an ‘adenocarcinoma-like’ profile [56]. The concordance between the ctDNA mutation rate and immunohistochemistry findings was 64% for TP53 and 14% for RB1. The patients in the pilot study with *KRAS* and *BRAF* mutations treated with platinum and etoposide chemotherapy and the patients with *RB1* mutations treated with folinic acid, 5-fluorouracil and irinotecan (FOLFIRI) chemotherapy had shorter PFS. This suggests that ctDNA analysis is sensitive and can be useful in patients with a diagnosis of NEC, but the full results from the study are still awaited.

Although ctDNA is promising as a liquid biopsy and can identify targetable mutations, this is yet to be validated. Further large scale studies are recommended to assess the role of ctDNA in the management of patients with a NEN diagnosis.

## 5. RNA

The transcriptome is responsible for gene expression and protein synthesis and consists of all RNA transcripts, coding and non-coding, in a cell at any given time [22]. The human genome encodes approximately 20,000 genes which are transcribed into messenger RNA (mRNA), ribosomal RNA (rRNA) and transfer RNA (tRNA) [22]. Abnormal expression of RNA is a feature in cancer and transcriptional profiling of tissue from tumours has identified transcripts associated with NETS that are detectable in the circulation. These have been identified as sources for detection in liquid biopsies [57].

### 5.1. Circulating mRNA

The majority of mRNA present in blood is susceptible to degradation by ribonuclease (RNase) and are labile in nature, resulting in variable detection [58]. Plasma mRNA markers have been investigated in several tumour types. One of the first studies assessing plasma mRNA determined that amplification of tumour mRNA from serum is feasible in patients with melanoma [59]. In breast cancer, the presence of cyclin D1 mRNA in the plasma of patients was associated with poor outcomes and non-response to tamoxifen [60]. The identification of mRNA in plasma of patients with cancer has resulted in the study of transcripts in order to develop panels to determine tumour gene expression.

There is currently limited evidence on the clinical utility and validity of circulating mRNA in patients with NENs. Commercial mRNA-based assays have been developed in order to assess tumour gene expression in NENs. The NETest is reported to be a multianalyte evaluation of mRNA transcripts related to NETs, present in blood. These transcripts are analysed to determine tumour gene expression using an algorithm to produce a numeric disease score [57]. Following isolation of circulating mRNA, complementary DNA (cDNA) is synthesised and polymerase chain reaction (PCR) is performed against 51 targeted genes that are reported to capture the genomic profile of NET cells [61]. A diagnostic score is generated by determining the expression of genes in clusters and is presented as a clinical activity score ranging from 0% (low activity) to 100% (high activity) [57]. Several studies assessing the use of the NETest in diagnosis, monitoring disease and assessing treatment in patients with NENs have demonstrated favourable results [62,63,64]. However, its use has not yet been independently or externally validated in a randomised setting, and so caution is recommended in interpretation of these results. Furthermore, although individual gene testing for cancers offers the potential for targeted treatment, panels assessing multiple genetic markers to generate a risk score have yet to demonstrate its value as surrogate endpoints for disease.

### 5.2. Micro RNA

Micro RNA (miRNA) consists of short non-coding RNA (22 nucleotides) involved in regulation of gene expression [65]. Significant dysregulation in miRNA expression has been noted in cancer cells and this can cause proliferative signaling in cells, evasion of suppressor regulation and avoidance of apoptosis [65]. Their stability in blood and potential to discriminate different cancer types has generated interest as a potential biomarker [66].

Their role in the management of patients with NENs, however, remains uncertain, as miRNA has been implicated as both oncogene and tumour suppressors [67]. Small studies have generally assessed circulating miRNA expression in patients with GEP-NETs and have found dysregulation of miR-21in patients with small bowel and pancreatic NETs, miR-222 in gastric NETs and miR-7-5p in small bowel NETs [68,69,70]. However, there has been a lack of concordance between circulating miRNA expression and tumour miRNA.

Challenges exist in assessing miRNA, as there is no accepted measurement standard in studies. Current evidence is modest and there needs to be further understanding of the relevance of miRNA evaluation in patients with NENs.

## 6. Future Perspectives

**Figure 1:**
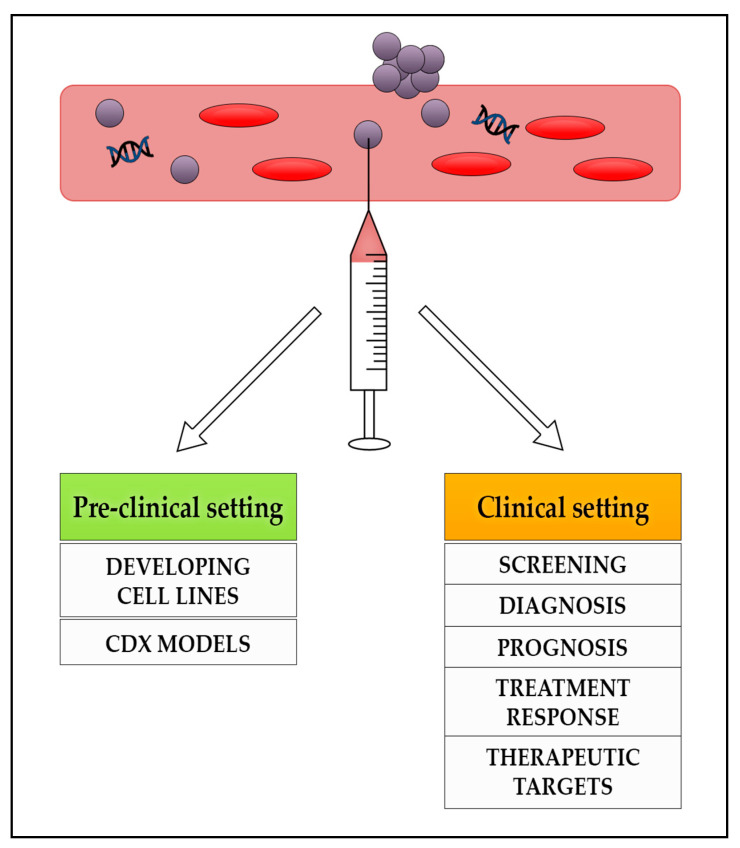
The potential role of liquid biopsies in the management of patients with NENs in the pre-clinical and clinical settings. NENs: neuroendocrine neoplasms; CDX: circulating tumour cells derived eXplant.

**Figure 2:**
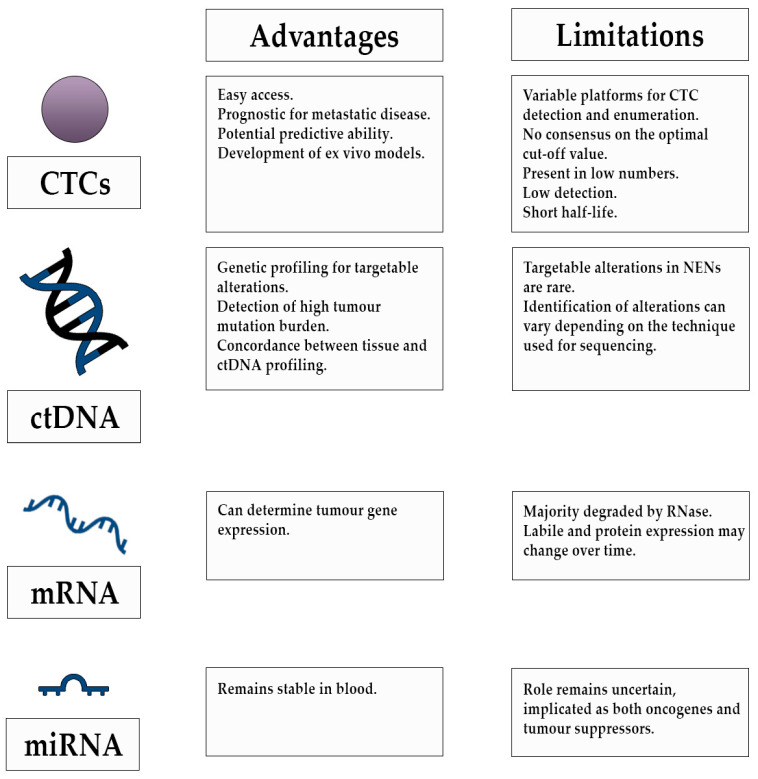
Advantages and limitations of circulating tumour marker use in the management of patients with NENs. CTCs: circulating tumour cells, ctDNA: circulating tumour DNA, mRNA: messenger RNA, miRNA: micro RNA, NENs: neuroendocrine neoplasms.

Existing biomarker uses in the management of patients with NENs have limitations and circulating tumour biomarkers have demonstrated promising results across various cancer types. It has become increasingly evident that liquid biopsies are superior in allowing real time disease management and reducing the need for single time point tumour tissue sampling, which can be difficult to access and may not represent the heterogeneity of the disease. Figure 1 highlights the potential use of liquid biopsies in the management of patients with NENs in the preclinical and clinical setting and Figure 2 highlights the advantages and limitations of their use.

### 6.1. Diagnosis

The use of CTCs as a diagnostic maker in patients with NENs is highly attractive, as it provides easy access and has the potential to reduce costs. However, most data on the presence of CTCs are mainly in the metastatic setting, which hinders use as a biopsy. Evidence from other tumour types such as prostate cancer has demonstrated potential use in locally advanced disease; however, CTCs appear to be present in a lower number of patients with localised disease [71]. Although CTC counts have been associated with all cancer stages and grades in patients with NENs, it is implicated predominantly in metastatic disease [29]. An effective liquid biopsy for a diagnosis of NENs would also require CTCs to be detectable in early stage disease. In addition, the low detection rates in this tumour type impairs potential clinical application. Research goals for future studies of CTCs in NENs should aim to also include patients with resectable and early stage disease in a prospective setting to assess their value as a diagnostic marker.

### 6.2. Prognosis

Circulating tumour cells have widely been associated with poor outcomes, cancer recurrence and metastases, and their main use may be in determining prognosis in patients with NENs. Studies have demonstrated that low baseline counts are associated with better PFS and OS, and high CTC counts are associated with progressive disease and metastases. The main limitations of CTCs are that they are rare and only present in blood in low numbers, with less than 0.01% of cells released surviving to produce metastases [72,73]. Their detection rate is generally low and their half-life in the bloodstream is short, and as a result their use in clinical practice could be limited [72,73]. Research should focus on additional methods for enumeration and enrichment of CTCs, as CellSearch is the only currently approved platform for analysis, and other potential methods may yield higher detection rates of CTCs that have low or down-regulated expression of EpCAM. In addition, further characterisation of these cells may help to further the understanding of the biology behind the metastatic process. Circulating tumour cells are yet to be evaluated as a Type II biomarker (surrogate endpoints for disease that predict clinical benefit), and studies are needed to assess the utility of CTC assessment in the management of patients with NENs, with greater emphasis on its value in clinical decision making.

### 6.3. Predictive

Circulating tumour cells have shown promise as a predictive marker for radiological response to treatment and evaluating SSTR status in patients with NENs [30,39]. The predictive value of CTCs in NENs, however, needs further assessment, to establish its clinical relevance. Since evidence to date has been modest regarding the clinical use of these approaches in the management of patients with NENs, attention has turned to the use of transcriptional profiles as a predictive marker [57]. However, it is worth noting that, due to the dynamic nature of NENs, levels of RNA transcripts and protein expression may change over time and any test may not encompass all relevant alterations, potentially limiting widespread use.

### 6.4. Development of Ex-Vivo Models

CTC-derived eXplant models can be of use in preclinical settings to help shape the understanding of tumour biology in patients with NENs and test novel therapeutic targets. Circulating tumour cell-derived mouse models can be used in patients unable to undergo tissue sampling and can also be collected and implanted at different time points, allowing models to capture temporal and spatial tumour heterogeneity. Currently, CDX models generated from NENs are scarce and further development is a prospect for future studies.

### 6.5. Targeted Treatment and Study of Resistance Mechanisms

Current treatment for patients with a NEN diagnosis has not yet been tailored to select patients on the basis of molecular alterations. Circulating tumour DNA analysis has been shown to be feasible and demonstrated promise in identifying therapeutic targets in patients with NENs according to several case reports. Furthermore, the analysis of ctDNA may be useful in determining high tumour mutation burden. Circulating tumour DNA contains genetic mutations that are identical to the original tumour cells; however, its detection and use is limited by the lack of targetable alterations in NENs. In addition, concordance between tissue and plasma do vary between studies and may reflect the technique used for sequencing, or the varying composition of the tumours [50]. Current evidence of the utility of ctDNA in the management of patients with NENs is at an early stage, and it is worth noting that pathological alterations are more commonly detected in NECs than in well-differentiated disease. Future studies of ctDNA warrant further in depth analysis in this particular patient group (NECs) in order to identify potential therapeutic targets.

Circulating tumour cells have been reported to develop mutations following release into the circulatory system from the primary tumour sites, and may be responsible for the development of uninhibited survival and resistance to treatment of these cells [74]. Molecular profiling of CTCs can help further the understanding of the underlying disease processes and mechanisms behind resistance [74]. This has been observed in metastatic breast cancer, where a ‘CTC-Endocrine Therapy Index’ was developed to assess the feasibility of determining CTC expression of four markers associated with endocrine therapy failure [75]. A high score was hypothesised, by the authors, to be associated with treatment resistance and therefore, unlikely to gain clinical benefit. Although, relatively unexplored in NENs, further study of CTCs implicated in treatment resistance may help contribute to development of personalised treatments for patients.

### 6.6. Clinical Trials

Several trials assessing the use of liquid biopsies in patients with NENs are currently underway.

NET 02 is an ongoing multi-centre randomised phase II study (NCT03837977) assessing the use of liposomal irinotecan and 5-fluorouracil/folinic acid or docetaxel as second line therapy in patients with poorly differentiated extrapulmonary NEC. In addition to assessing treatment response, the study will analyse CTCs and ctDNA to assess for correlation with patient outcomes. Mouse models will also be generated to help further the understanding of the tumour biology [76]. NCT01744249 is a Phase II/III, prospective, randomised study assessing the efficacy of axitinib in patients with advanced nonpancreatic NETs. The phase III part of the study will compare Sandostatin LAR with axitinib to Sandostatin LAR with placebo. CTCs, circulating endothelial cells and mRNA transcripts will be analysed to assess their predictive value. Another study, (NCT02973204) investigates CTCs and ctDNA in patients with hepatocellular carcinoma and NET in order to identify tumour specific mutations.

The results from these studies are anticipated to add to the growing evidence of the role of liquid biopsies in the management of patients with NENs.

## 7. Conclusions

The complexity in the biological behaviour of neuroendocrine neoplasms cannot be accurately measured with conventional biomarkers. Tumour tissue sampling for diagnosis has limitations and liquid biopsies can adequately capture spatial and temporal intra-tumour heterogeneity needed to make clinical decisions. Tumours from patients with NENs are best assessed via a multidimensional approach, focusing on the detection of mutations and gene expression, in addition to analysis of ctDNA and mRNA and exploration of their potential applicability in identifying targetable treatments and treatment response. In addition, evaluating additional methods for CTC detection may help enhance the field of research in relation to the potential use of CTCs as surrogate endpoints for disease and CDX models for tumour characterisation. Future studies should focus on assessing the application of CTCs, ctDNA and mRNA, in conjunction with current clinical practice, in prospective cohorts to inform personalised care in patients with NENs.

## Figures and Tables

**Table 1:** Summary of relevant evidence of potential utility of circulating tumour cells (CTCs) in patients with neuroendocrine neoplasms (NENs).

Author	Tumour Type	N	Biomarkers	CTC Cut-Off Value	CTC Detection	Outcomes
Hseih et al., 2019 [29]	Unresectable locally advanced and metastatic NENs	35	CTCs (EpCAM independent)	Three cut-offs: ≥1, ≥5, ≥20 cells/mL blood)	43% had detectable CTCs	CTC counts associated with cancer stages (I-III vs. IV, *p* = 0.015), liver metastasis (*p* = 0.026), and NET grading (*p* = 0.03). Baseline CTC counts prognostic factors for PFS survival (*p* = 0.015) and OS (*p* = 0.023).
Rizzo et al., 2019 [38]	Metastatic bronchial, midgut or pancreatic NENs	254	CTCs with CXCR4 expression EpCAM +ve	≥1 CTC 7.5/mL blood	43% had detectable CTCs	Bone metastases significantly associated with CTCs (*p* < 0.0001) CXCR4-positive CTCs in patients with bone metastases was 56% compared to 35% in those without (*p* = 0.18)
Khan et al., 2016 [30]	Metastatic NENs commencing therapy	138	CTCs with EpCAM expression	≥1 CTC 7.5/mL blood	68% had detectable CTCs.	Changes in CTCs had strong association with OS (HR, 4.13; *p* = 0.0002). Better prognosis in patients with 0 CTCs before and after therapy; followed by those with ≥50% reduction in CTCs (HR 3.31) Poor outcomes in patients with a <50% reduction or increase in CTCs (HR, 5.07).
Childs et al., 2016 [39]	Metastatic midgut, pancreatic or CUP NETS	31	CTCs with SSTR expression EpCAM +ve	≥1 CTC 7.5/mL blood	68% had detectable CTCs	33% had expression of SSTR2/SSTR5 87% (n = 27) of all patients had SSTR-positive tumours according to somatostatin receptor scintigraphy or 68Ga PET CT
Khan et al., 2013 [28]	Metastatic NENs	175	CTCs with EpCAM expression	≥1 CTC per 7.5 mL	49% patients had ≥ one CTC, 42% had ≥ two CTCs, and ≥ 30% had five CTCs	≥one CTC associated with worse PFS and OS (hazard ratios [HRs], 6.6 and 8.0, *p* < 0.001). CTCs associated with poor prognosis. Grade 1, HRs were 5.0 for PFS (*p* < 0.017) and 7.2 for OS (*p* < 0.023); Grade 2, HRs were 3.5 for PFS (*p* < 0.018) and 5.2 for OS (*p* < 0.036).
Khan et al., 2011 [27]	Metastatic NENS	74	CTCs with EpCAM expression	NR	43% of midgut and 21% of pancreatic NETs had detectable CTCs 68% > 5 CTCs	Absence of CTCs strongly associated with stable disease (*p* < 0.001) Moderate correlation between CTC levels and burden of liver metastases (B = 8.91, *p* < 0.001)

CTCs: circulating tumour cells, NETs: neuroendocrine tumours, NEC: neuroendocrine carcinoma, HR: hazard ratio, PFS: progression free survival, OS: overall survival, EpCAM: epithelial cell adhesion molecule, SSTR somatostatin receptor, CUP: cancer of unknown primary.

**Table 2:** Summary of available evidence of potential utility of circulating tumour DNA (ctDNA) in patients with neuroendocrine neoplasms (NENs).

Author	Tumour Type	N	Biomarkers	Outcome	Clinical Relevance
Zakka et al., 2020 [50]	Pancreatic NET, gastrointestinal NEC, large cell lung NEC, nasopharyngeal NEC	320	ctDNA analysis	Genomic alterations found in 87.5% of samples Total of 1012 alterations identified Mutations in TP53 52%, KRAS, 22%, EGFR 12%, PIK3CA,11%, BRAF 10%, MYC 10%, CCNE1 10%, CDK6 8%, RB1 7%, NF1 7%, MET 7%, FGFR1 7%, APC 7%, ERBB2 6% and 5%.	Evaluation of ctDNA was feasible in NENS and may help determine driver mutations for targeted therapy
Wang et al., 2017 [51]	Metastatic atypical carcinoid tumour of the lung	1	ctDNA analysis	ctDNA analysis revealed ALK translocation Treated with ALK inhibitor alectinib with partial response. Approximately 60% shrinkage of dominant brain metastases	ctDNA is a feasible alternative platform for identifying driver mutations when tissue sampling is limited. It may help determine targeted therapy
Boons et al., 2018 [52]	Pancreatic NET undergoing surgery	10	cfDNA analysis	Tumor-specific variants were detected in 2 PNET patients, at variant allele fractions of 19% and 21%. In the metastatic patients, there was correlation between copy number variations of tumour tissue profiles and cfDNA.	Copy number variation analysis in cfDNA has potential as a liquid biopsy
Beltran et al., 2020 [53]	Castration-resistant neuroendocrine prostate cancer (CRPC-NE)	17	cfDNA and ctDNA analysis	High concordance between cfDNA and biopsy tissue genomic alterations Mutations found in *RB1* (69%) and *TP53* (63%) in CRPC-NE patients. Prior exposure to cytotoxic chemotherapy was associated with higher cfDNA	Evulation of cfDNA is feasible in CRPC-NE and may help determine genomic changes associated with the disease
Sharabi et al., 2017 [54]	High-grade, large-cell neuroendocrine carcinoma of the cervix	1	ctDNA analysis	Multiple alterations in ctDNA suspicious for high tumour mutational burden. Nivolumab commenced on the basis of ctDNA results as tumour tissue awaited Tissue biopsy confirmed mismatch repair gene defect, concordant with ctDNA.	Evaluation of ctDNA is feasible and may help determine driver mutations for targeted therapy

NET: Neuroendocrine tumour, NEC: Neuroendocrine carcinoma, ctDNA: circulating tumour DNA, cfDNA: cell free DNA, PNET; pancreatic neuroendocrine tumours.
